# Severe dengue categories as research endpoints—Results from a prospective observational study in hospitalised dengue patients

**DOI:** 10.1371/journal.pntd.0008076

**Published:** 2020-03-04

**Authors:** Kerstin D. Rosenberger, Neal Alexander, Eric Martinez, Lucy C. S. Lum, Carl-Erik Dempfle, Thomas Junghanss, Bridget Wills, Thomas Jaenisch, the DENCO Clinical Study Group

**Affiliations:** 1 Section Clinical Tropical Medicine, Department for Infectious Diseases, Heidelberg University Hospital, Heidelberg, Germany; 2 Department of Infectious Disease Epidemiology, London School of Tropical Medicine and Hygiene, London, United Kingdom; 3 Pedro Kouri Institute for Tropical Medicine, Havana, Cuba; 4 Department of Paediatrics, University of Malaya, Kuala Lumpur, Malaysia; 5 IMD Coagulation Center Mannheim, Mannheim, Germany; 6 Oxford University Clinical Research Unit, Hospital for Tropical Diseases, Ho Chi Minh City, Vietnam; 7 Centre for Tropical Medicine and Global Health, Nuffield Department of Clinical Medicine, Oxford University, Oxford, United Kingdom; University of Heidelberg, GERMANY

## Abstract

Severe dengue was perceived as one clinical disease entity until the WHO 2009 classification stratified it into severe vascular leakage, severe bleeding, and severe organ dysfunction. The objectives of this study were to investigate the potential use of severe dengue categories as endpoints for intervention research. 271 patients with severe dengue among 1734 confirmed dengue patients were followed prospectively in this hospital-based observational study in Latin America and Asia. We compared the distribution of severe dengue categories according to gender and age (below/above 15y), and determined the relative frequency and the overlap of severe dengue categories in the same patients. In a next step, we extended the analysis to candidate moderate severity categories, based on recently suggested definitions which were adapted for our purposes. Severe vascular leakage occurred in 244 (90%), severe bleeding in 39 (14%), and severe organ dysfunction in 28 (10%) of 271 severe dengue patients. A higher frequency of severe leakage was seen in children or adolescents (<15y) compared to adults. More than 80% of the severe leakage cases, and 30–50% of the cases with severe bleeding or severe organ dysfunction, were defined as severe on the basis of that feature alone. In 136 out of 213 patients with severe leakage alone, neither moderate bleeding manifestation nor hepatic involvement was recorded. On the other hand, moderate leakage manifestations were detected in 4 out of 12 cases that were classified as severe based on bleeding alone. A major proportion of severe dengue patients exhibited clinical manifestations of severe vascular leakage only, which may constitute a useful endpoint for intervention research or pathophysiology studies. Severe bleeding and severe organ manifestation were recorded less frequently and exhibited a higher degree of overlap with severe leakage. Severe bleeding without leakage may be associated with individual predisposition or the presence of comorbidities. More detailed assessments are needed to explore this hypothesis. Candidate moderate disease endpoints were investigated and need to be further validated.

## Author summary

Severe dengue (DHF/DSS) was perceived as one clinical disease entity until the WHO 2009 classification defined three categories: severe vascular leakage, severe bleeding, and severe organ dysfunction. Here we provide new insight about the discriminatory power and the overlap or co-occurrence of the severe dengue categories. We also respond to the need to validate standard clinical endpoints for severe dengue in well-characterized data sets. In addition, we respond to the need for moderate disease endpoints, which are recorded more frequently than the severe ones. Severe vascular leakage alone constitutes the largest group among the severe dengue categories and may be useful as endpoint for intervention or pathophysiology research. Other severe dengue categories as for example severe bleeding can also sometimes occur alone–however, this might be associated with the presence of comorbidities or individual predisposition and should be studied in more detail. Moderate disease endpoints were suggested and explored here, but need to be further investigated.

## Introduction

Dengue is one of the most important mosquito-borne diseases to affect humans worldwide. It is known to be present in 126 countries, where 2.5 billion people or 40% of the world’s population are at risk [1], and an estimated 390 million infections occur annually, of which around 100 million are symptomatic [2].

The clinical spectrum ranges from mild febrile illness to severe and potentially life-threatening disease [3]. In accordance with the World Health Organization (WHO) 2009 dengue classification scheme [4], the severe end of the disease spectrum is defined as: i) severe vascular leakage resulting either in dengue shock syndrome or in respiratory distress due to fluid accumulation; ii) severe bleeding; and iii) severe organ dysfunction. These severe categories appear to affect children and adults differently [5]; thus severe vascular leakage is typically observed more frequently in children, and severe bleeding more frequently in adults [6]. However, the extent to which these severity categories overlap in the same patients remains unclear. As the geographical spread of dengue has increased globally, severe disease is now being reported in populations of all ages and with varying immunological backgrounds, resulting in a diversification of the clinical picture. At the same time, there is an urgent need for operationalisation and standardisation of clinical endpoints for dengue research studies.

In a recent effort to define standard clinical endpoints for use in dengue intervention trials, criteria for moderate disease severity as well as severe disease endpoints were suggested by an expert group, using a structured Delphi consensus method. The authors conclude by saying that the endpoints suggested should be validated, ideally using existing large prospective datasets [7].

Between 2006 and 2007 we performed a prospective multicentre observational study (the DENCO acute febrile illness cohort study) that enrolled participants with suspected dengue in 7 countries across Asia and Latin America [8], resulting in one of the largest and best curated datasets currently available describing the severe end of the clinical spectrum in a substantial number of hospitalised dengue patients. Results from this study provided evidence to support the 2009 revision of the WHO dengue case classification based on clinical severity [8, 9]. The objective of the analysis presented here was to respond to the need for validation of standardized endpoints, and to investigate the potential of the severe dengue categories to represent distinct clinical endpoints for intervention or pathophysiology research in dengue. This also led to further exploration of the candidate definitions for moderate severity, which were adapted from the definitions recently suggested by the expert group [7], considering the availability of the proposed criteria/laboratory information in the DENCO dataset.

## Methods

### Study design & patient population

In the observational DENCO cohort study a total of 2259 patients with a possible diagnosis of dengue were enrolled at 11 hospitals across seven dengue endemic countries. Trained physicians used a single comprehensive case report form to follow all study participants daily throughout the evolution of their illness. Details of the study design, patient population, classification algorithms, clinical definitions and laboratory criteria for diagnosis of dengue have been described elsewhere [8]. Ethical approval was obtained from the review boards of each hospital where patients were enrolled and from the Ethics Review Committee of WHO, and written consent was provided by each participant or a parent/guardian prior to study enrolment. Clinical monitoring was carried out by WHO-trained monitors following ICH-GCP guidelines.

A total of 1734/2259 (77%) patients enrolled in the study were confirmed as having dengue in accordance with a pre-specified diagnostic algorithm [8]. Demographics of the confirmed dengue patients are described in **Table 1.** Of these, 271 were classified as having severe dengue, following the system subsequently adopted in the 2009 WHO classification and summarised in **Table 2** [4]; in total 244 patients were classified as severe by vascular leakage, 39 by severe bleeding and 28 by severe organ dysfunction, with more than one severe category identified in 32 of the cases. We first concentrated on the overlap of the categories at the severe end of the clinical spectrum. In a second step, we expanded the analysis of the overlap beyond severe disease and included less severe measures of vascular leakage, bleeding, and organ dysfunction in the severe patients, using the “moderate severity” definitions presented in **Table 2**, which were adapted from the definitions recently suggested by Tomashek et al. [7]. The reason for adapting the definitions was that this data set was collected before these definitions became available and therefore did not include certain information as for example if local interventions for bleeding (e.g. pressure package) or cross-matching for blood transfusion were carried out.

**Table 1:** Characteristics of study population.

	Non-severe Dengue	Severe Dengue
**Age**	13 (9–20)	12 (8–17)
**Sex** **Female** **Male**	668 (82%) 795 (86%)	142 (18%) 129 (14%)
**Country** **Brazil** **Nicaragua** **Venezuela** **All Latin America** **Malaysia** **Philippines** **Thailand** **Vietnam** **All Southeast Asia**	32 (91%) 71 (72%) 91 (92%) 194 (83%) 116 (72%) 308 (81%) 137 (82%) 708 (89%) 1269 (85%)	3 (9%) 28 (28%) 8 (8%) 39 (17%) 45 (28%) 72 (19%) 30 (18%) 85 (11%) 232 (15%)

Numbers represent the frequency (%) for binary and categorical variables and the median (interquartile range) for continuous variables.

**Table 2:** Severe Dengue (SD) according to the WHO 2009 dengue classification, and moderate dengue according to the definitions used for this analysis.

	Severe Dengue (SD)	Moderate dengue severity
**Vascular leakage**	- Shock (DSS) - Fluid accumulation with respiratory distress	- Serosal fluid accumulation*
**Bleeding**	- As evaluated by clinician, leading to haemodynamic instability**	- Mucosal bleeding (excluding uncomplicated haematuria***) without haemodynamic instability
**Organ dysfunction**	- Liver: AST or ALT ≥ 1000 U/L - CNS: Impaired consciousness (coma scale < 15 [GCS] or < 5 [BCS])	- Liver: 400 U/L ≤ ALT < 1000 U/L

* Serosal fluid accumulation was diagnosed either clinically or radiologically. Clinical fluid accumulation was defined as clinical pleural effusion or ascites on any day. Radiologically detected fluid accumulation (assessed within 24 hours of defervescence) was defined as pleural effusion by CXR or serosal fluid collection on ultrasound; see also Rosenberger et al., 2016 [33].

** Bleeding cases experienced gastrointestinal, skin or mucosal bleeding (e.g. nose bleed / bleeding at venepuncture sites; often multiple sites), which was evaluated as ‘severe’ by the treating physician based on the concern that the bleeding lead to haemodynamic instability. In most cases, severe bleeding was followed by administration of blood products.

*** Haematuria was defined either microscopically / dipstick test (≥10 erythrocytes/μl), or as macroscopic haematuria.

### Statistical analysis

The distribution and overlap of the three severe categories were analysed using frequency distributions (including 95% CI of proportions) and Venn diagrams, stratified by age group (<15years vs. ≥15years), gender, and continent (Latin America vs. Asia).

To assess whether laboratory variables and clinical signs or symptoms differed between the severe dengue categories we carried out linear discriminant analysis (LDA). We evaluated linear combinations of variables, defined by coefficients (loadings) that best distinguished between the severe leakage and severe bleeding categories [9]. As the severe organ dysfunction category was relatively small and at the same time more heterogeneous (comprising a liver and a CNS subgroup) than the bleeding category we elected not to include this category in this comparative analysis. The initial list of candidate variables included 21 clinical (binary) and 6 laboratory (continuous) variables (**S1 Table**). Most of these explanatory variables are binary, but multivariate normality is not a requisite for LDA to perform well [10]. Variable selection was based on the criteria of (i) at least 90% non-missing values, (ii) ≥ 5% abnormal values, and (iii) retaining only one of the variables with similar content (e.g. ‘liver enlarged by more than 2cm’ and ‘liver palpable’). Finally, 13 binary and two continuous candidate variables were chosen, their values being taken from the day when the criteria for either severity category were first met [11]. The binary variables selected were nausea, abdominal pain or tenderness, lethargy, liver enlargement by more than 2cm, skin flush, rash, oedema, clinical pleural effusion, ascites, mucosal bleeding, skin bleeding, haematocrit 20% over the baseline, and thrombocytopenia below 100,000 cells/μl. Haematocrit increase and platelet count were also included as continuous variables. For the baseline haematocrit we collated data from healthy individuals stratified by continent, age, and sex as previously described [8]. We aimed to discriminate between the groups using the smallest number of explanatory variables. Therefore, we initially included all the parameters listed above, subsequently proceeding to drop first those with negative adjusted *R*^2^ from the model, then, one by one, those with *p* values more than 0.05.

For the LDA, patients diagnosed as severe in more than one category were classified according to the first severe category they reached during the evolution of the illness episode. For participants who qualified for both the severe leakage and severe bleeding categories on the same day we performed the analysis twice, once assigning them to severe vascular leakage, and again assigning them to severe bleeding. All data were analysed using STATA versions 10 or higher (STATA Corporation, College Station, Texas).

## Results

### Epidemiology of severe dengue

Among the 271 patients classified as severe, 184 were below 15 years of age (17% of all patients < 15 years; 95% CI 15.1–19.7) while 87 were aged 15 or more (13% of all patients ≥ 15 years; 95% CI 10.5–15.7). The majority of the severe patients were enrolled in Asia (N = 232; 86%) compared to Latin America (N = 39), but the relative proportion of severe patients as well as the relative frequency of severe dengue categories did not differ between the continents (**Table 3**).

**Table 3:** Frequency of severe dengue categories comparing children (< 15 years) versus adults and Asia versus Latin America.

	Overall	Asia & Latin America		Asia		Latin America	
		**< 15 years**	**> = 15 years**	**< 15 years**	**> = 15 years**	**< 15 years**	**> = 15 years**
**Non-Severe**	1463 (84.4%; 82.1–86.1)	878 (82.7%; 80.3–84.9)	585 (87.1%; 84.3–89.5)	745 (83.0%; 80.4–85.5)	524 (86.8%; 83.8–89.4)	133 (80.6%; 73.7–86.3)	61 (89.7%; 79.9–95.8)
**Severe** Male* Female*	271 (15.6%; 13.9–17.4) 129 (14.0%; 11.8–16.4) 142 (17.5%; 15.0–20.3)	184 (17.3%; 15.1–19.7) 88 (15.8%; 12.8–19.1) 96 (19.1%; 15.7–22.8)	87 (12.9%; 10.5–15.7) 41 (11.2%; 8.2–14.9) 46 (15.0%; 11.2–19.5)	152 (17.0%; 14.5–19.6) 71 (15.1%; 12.0–18.7) 81 (19.0%; 15.4–23.0)	80 (13.2%; 10.6–16.2) 41 (12.5%; 9.1–16.6) 39 (14.1%; 10.2–18.7)	32 (19.4%; 13.7–26.3) 17 (19.3%; 11.7–29.1) 15 (19.5%; 11.3–30.1)	7 (10.3%; 4.2–20.1) 0 7 (24.1%; 10.3–43.5)
Plasma Leakage** Clinical shock*** Fluid accumulation with respiratory distress***	244 (90.0%; 85.8–93.3) 210 (77.5%; 72.0–82.2) 79 (29.2%; 23.8–35.0)	**173 (94.0%; 89.6–97.0)** 147 (79.9%; 73.4–85.4) **68 (37.0%; 30.0–44.4)**	**71 (81.6%; 61.8–81.5)** 63 (72.4%; 61.8–81.5) **11 (12.6%; 6.5–21.5)**	144 (94.7%; 89.9–97.7) 120 (78.5%; 72.6–85.1) **55 (36.2%; 28.6–44.4)**	71 (88.8%; 79.7–94.2) 63 (78.8%; 68.2.-87.1) **11 (13.8%; 7.1–23.3)**	29 (90.1%; 75.0–98.0) 27 (84.4%; 76.2–94.7) 13 (40.6%; 23.7–59.4)	0 0 0
Bleeding**	39 (14.4%; 10.4–19.1)	22 (12.0%; 7.6–17.5)	17 (19.5%; 11.8–28.4)	19 (12.5%; 7.7–18.8)	13 (16.3%; 8.9–26.2)	3 (9.4%; 19.8–25.0)	4 (57.1%; 18.4–90.1)
Organ Dysfunction**	28 (10.3%; 7.0–13.6)	24 (13.0%; 8.5–18.8)	12 (13.8%; 7.3–22.9)	12 (7.9%; 4.1–13.4)	9 (11.3%; 5.3–20.3)	4 (12.5%; 3.5–29.0)	3 (42.9%; 9.9–81.6)
**Total**	1734	1062	672	897	604	165	68

Numbers represent frequencies (%; 95% confidence interval).

Numbers in the severity categories include overlapping cases.

Numbers in bold indicate statistically significant differences between age groups (non-overlapping 95% CI).

* Percentages refer to the number of male / female patients for the respective category in the denominator.

** Percentages refer to the overall number of severe patients in the denominator.

*** Percentages in the severe categories refer to the number of severe patients for the respective category in the denominator.

We observed a trend towards higher frequency of severe disease in females, particularly in Asian children, among whom 71/470 boys (15%; 95% CI 12.0–18.7) were diagnosed with severe disease compared to 81/427 girls (19%; 95% CI 15.4–23.0). In Latin America, case numbers were smaller and no difference was observed between male and female children, but all severe disease in adults was recorded in females (N = 7; 24% of all adult females in LA; 95%CI 10.3–43.5) (**Table 3**).

### Severe dengue categories

Severe vascular leakage occurred in 244 (90%), severe bleeding in 39 (14%), and severe organ dysfunction in 28 (10%) of 271 severe dengue patients. The severe dengue categories were associated with age; a higher frequency of vascular leakage was seen in children and a higher frequency of severe bleeding in adults. Among 184 children with severe dengue, 173 (94%; 95%CI 89.6–97.0) were diagnosed with severe vascular leakage compared to 71/87 (82%; 95% CI 61.8–81.5%) adults. In contrast, severe bleeding was observed in 17/87 adults (20%; 95% CI 11.8–28.4%) versus 22/184 (12%; 95% CI 7.6–17.5%) children (**Table 3**).

**Fig 1:**
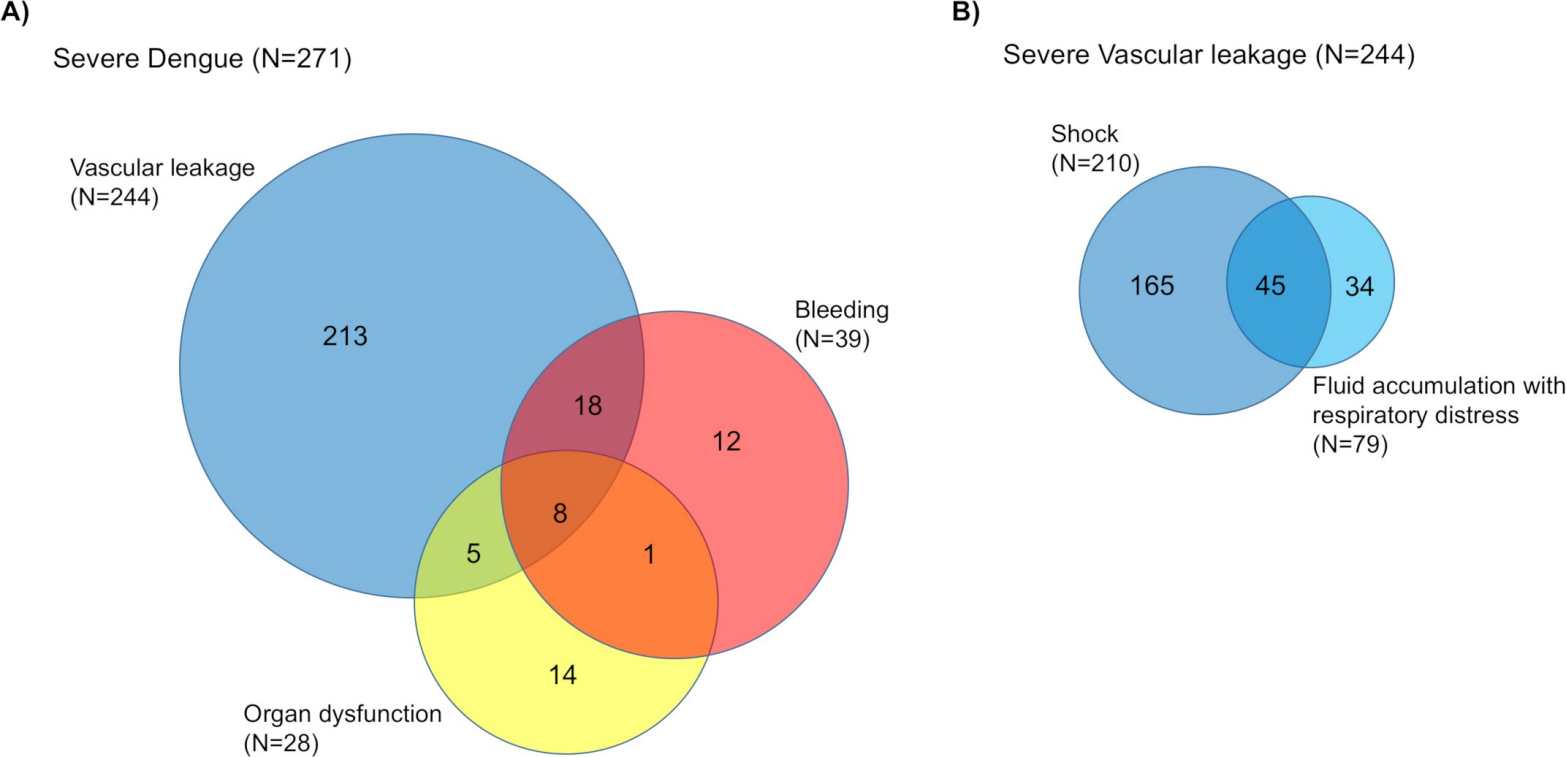
Overlap between severe vascular leakage, severe bleeding and severe organ dysfunction in 271 patients with severe dengue. (A) Overlap between severe plasma leakage, severe bleeding and severe organ dysfunction; (B) Overlap of shock and fluid accumulation with respiratory distress in 244 patients with severe vascular leakage.

The majority of severe cases was classified as severe in only one category (239/271, 88%), primarily represented by vascular leakage (**Fig 1**). The 239 patients who were diagnosed as severe on the basis of one category alone were further investigated regarding the presence of moderate severity in the other categories (**Table 4**).

**Table 4:** Presence of moderate disease severity in 239 severe dengue patients who qualified for severe disease in one clinical category only.

	N	Moderate severity N (%)			No moderate severity
		Serosal fluid accumulation*	Mucosal bleeding*	Liver (ALT ≥ 400 U/L)*	
**Vascular leakage only** Clinical shock* Fluid accumulation with respiratory distress*	213 182 66	-	72 (33.8%) 62 (34.1%) 19 (28.8%)	7 (3.3%) 5 (2.7%) 2 (3.0%)	**136 (63.8%)** **117 (64.3%)** **45 (68.2%)**
**Bleeding only**	12	4 (33.3%)	-	2 (16.7%)	4 (33.3%)**
**Organ dysfunction only**	14	10 (71.4%)	4 (28.6%)	-	**2 (14.3%)**

* Numbers include overlapping cases.

** 4 patients were unclassifiable for serosal fluid accumulation due to missing information, see **S2 Table**.

### Moderate severity categories

Among the 213 patients with severe leakage alone, mucosal bleeding as a candidate moderate bleeding marker occurred in 72 (34%), while hepatic involvement with moderately elevated ALT liver enzyme was observed in 7 patients (3%; 2 of 7 overlapping with mucosal bleeding) (**Table 4**). Thus, in the remaining 136 patients classified as severe by vascular leakage alone (64%), no moderate severity markers for bleeding or organ dysfunction were identified.

Twelve patients were classified as severe based on severe bleeding alone, most of them young adults (median age 21 years, IQR 14.5–26.5) who experienced severe bleeding from the nose or gastrointestinal tract, or at venepuncture sites. The majority received blood products. In four of these cases moderate vascular leakage was detected (i.e. clinical or radiological fluid accumulation without respiratory distress), and in another four patients the assessment was incomplete, due to lack of radiological diagnostic information within the pre-specified time-frame. Thus, four patients remain with severe bleeding, without any vascular leakage documented (**S2 Table**) as well as without any other moderate severity features (**Table 4**), while two of the 12 patients (17%) had moderate liver involvement.

In the 14 patients with severe organ dysfunction alone (6 with severe liver involvement and 8 with impaired consciousness), the majority (12/14, 86%) also had evidence of other moderate severity markers—10 had fluid accumulation, 4 had mucosal bleeding, among them 2 were diagnosed with both fluid accumulation and mucosal bleeding (**Table 4**).

### Association patterns between clinical and laboratory markers by severity category

We used linear discriminant analysis (LDA) to describe association patterns between clinical and laboratory markers, investigating the question whether severe categories can be distinguished by the profile of abnormalities. For this purpose, we concentrated on severe vascular leakage and severe bleeding occurring on the same day. After excluding individuals with missing values, we performed linear discriminant analysis in 222 patients. Within this subgroup, 13 patients met the criteria for severe bleeding, 201 for severe vascular leakage, and 8 met the criteria for both on the same day. Classifying these 8 individuals (with vascular leakage and bleeding) together with severe bleeding (total 21 cases), the LDA yielded a single discriminant function including the following explanatory variables: lethargy (loading 1.40); flush (-0.84); rash (-0.68); mucosal bleeding (-2.26); and skin bleeding (-0.40). Lethargy therefore had a positive loading (associated with leakage) while the others were negative (associated with bleeding). Twenty of the 21 individuals in the bleeding group (95%) and 155 of 201 individuals in the leakage group (77%) were correctly classified (**S1 Fig**).

When the 8 individuals were instead classified with the severe leakage group (total 209 cases), the variables flush, rash, and mucosal bleeding remained in the final model, still with negative loadings (associated with bleeding). Lethargy and skin bleeding on the other hand were replaced by clinical pleural effusion, ascites and haematocrit (in percent), all three associated with leakage. This correctly classified 11/13 (85%) of the bleeding group, and 170/209 (81%) of the vascular leakage group.

## Discussion

The data presented provide crucial insights regarding the relative frequency and overlap of the three severe dengue categories, vascular leakage, bleeding, and organ dysfunction. In the field of malaria research, a similar approach proved to be instrumental for the field when the overlap of clinical subgroups within the “severe malaria” umbrella was characterised, and prognostic features for these subgroups were identified [12]. However, the focus of the current analysis was to assess the potential utility of using severe and moderate dengue as endpoints for research studies, rather than on identifying prognostic features of these entities. The case fatality rate of hospitalised dengue cases is usually very low, estimated at <1% in many settings [4], but can be substantially higher if medical management is not appropriate [13]. Only 2 of 1734 confirmed dengue patients in this study died—both children with severe vascular leakage and shock—precluding use of these data for evaluation of prognostic factors relating to survival.

We were primarily interested in identifying differences between the disease categories, rather than trying to distinguish between severe and moderate disease in general. Previously, statistical evidence for a distinct overall moderate severity group has not been corroborated [8]. The moderate disease definitions per category used in this analysis (**Table 2**) were based on previous work using this data [33], as well as on the endpoints suggested recently by an expert working group [7]. However, some of the variables listed in their moderate disease endpoint definitions—e.g. need for local intervention for bleeding (e.g. pressure packing) or ‘crossmatch performed for potential blood transfusion’—were not available, even in this well-characterized prospective research dataset, and therefore the definitions had to be adapted.

The relative frequency of severe dengue categories differed according to age group. In line with previous reports, vascular leakage was significantly more frequently observed in children while bleeding manifestations occurred more frequently in adults, although this was not statistically significant [5, 6]. It has also been argued that dengue in the Americas differs from Asia [14], although we did not find a clear difference with this dataset.

With regard to gender, we saw a trend towards more severe disease in females in Asia, albeit not statistically significant, with this trend more pronounced in girls compared to adult females. Similar findings have been reported previously [15, 16], including higher mortality in girls in Asia, and have been attributed to differences in health seeking behaviour or potentially also to underlying differences in physiological responses between the sexes [17]. In Latin America, overall numbers were considerably smaller compared to Asia and the results should be interpreted with caution. However, we could not detect a signal for more severe disease in girls compared to boys in Latin America. Interestingly, among adults all 7 severe disease cases were female–in these instances not due to severe vascular leakage, but related to bleeding and organ dysfunction.

From a phenotypic point of view, more than 80% of the severe vascular leakage cases and 30–50% of the cases with severe bleeding or severe organ manifestations were classified as severe based on defining features present in only one severity category. Thus, severe leakage alone may constitute a useful endpoint for intervention research or pathophysiology studies. Using linear discriminant analysis, we were able to show that the group of patients with severe leakage could be distinguished, based on their clinical and laboratory profiles, from a second group that included most of the severe bleeding cases.

In a minority of cases we saw severe bleeding without vascular leakage documented. The literature describing dengue cases with severe bleeding, but without evident vascular leakage, has been summarized by Bandyopadhay et al. [18]. However, many of the existing reports were based on retrospective data where leakage manifestations might have gone unnoticed. In response to these reports investigators have suggested new terminology such as ‘DFB’ (DF with unusual bleeding in the Delhi outbreak of 1996) or ‘DF with haemorrhage’ [19]. The terminology of ‘DF with unusual hemorrhage’ was included in the PAHO guidelines in 1994 [20]. More recently, a series of dengue cases presenting with intracranial haemorrhage, but without evidence of severe leakage, were reported by Sam et al. [21].

In addition to thrombocytopenia, various derangements in haemostatic parameters have been described in association with dengue, including endothelial activation with release of von Willebrand-factor [22], moderate coagulation activation with formation of thrombin-antithrombin complexes, D-dimers and decreased fibrinogen levels [23], reduced thrombin formation and excessive fibrinolysis [24], possibly with plasminogen activation. Plasminogen has been shown to be activated by dengue virus in vitro [25] and plasminogen cross-reactive antibodies have been detected during infection [26, 27]. However, clinical bleeding manifestations are generally thought not to reflect a primary consumptive coagulopathy [23, 28], except when compounded by the effects of profound shock or secondary bacterial infection when true disseminated intravascular coagulation and major haemorrhage may occur.

In a disease like dengue with a broad clinical spectrum, research to elucidate mechanistic pathways underlying specific phenomena relies on careful characterisation of individual clinical phenotypes. The extent to which the original WHO 1997 and the revised 2009 dengue classification systems facilitate pathophysiology research has been a matter of some debate [29, 30]. For example, it has been argued that the 2009 severe dengue entity represents a mix of end-stage manifestations involving various clinical pathways and potentially including comorbidities and/or iatrogenic factors [31]. However, the majority of experts attending a consensus meeting in Latin America felt that the 2009 WHO classification is helpful in identifying cases of dengue correctly for pathophysiological research, although they also recommended ongoing research to improve the sensitivity and specificity of the definitions.[32].

Possible limitations of the current study include the fact that some participating hospital sites were paediatric institutions, which is one reason why age-stratified results are reported. Additionally, due to the observational nature of the study, medical management was not standardized, but all participating hospitals were local centres of excellence specialising in treatment of dengue patients using local protocols derived from the WHO management guidelines. We also acknowledge that the analysis was not stratified by serotype and that the majority of severe cases were diagnosed in Asia. However, the relative frequency of the severe categories within the age groups was comparable between Asia and Latin America. Furthermore, our results reflect the clinical epidemiology usually observed in dengue-endemic countries where the majority of severe cases exhibit secondary immune responses.

### Conclusions

With 271 severe dengue patients recruited under the same protocol, this observational cohort study represents one of the largest prospective datasets currently available describing the severe end of the clinical spectrum in hospitalised dengue patients. The majority of the severe dengue cases in this study were classified as severe based on defining features in only one severe category, primarily vascular leakage.

Our results suggest that severe vascular leakage without (severe) bleeding or organ manifestations can be reliably distinguished and therefore may be useful in itself as an endpoint for intervention research as well as for pathophysiology research.

In a minority of cases we saw severe bleeding without vascular leakage documented. If not caused by the heterogenous quality of assessment, we believe that this might result from a predisposition where dengue served as a trigger for bleeding complications. In this case, the bleeding is unlikely to be the result of consumption coagulopathy or hyperfibrinolysis, but rather caused by the derangements in the context of vascular leakage. Therefore, severe bleeding without leakage may constitute a pragmatic endpoint for intervention research—however, does not necessarily imply the existence of a distinct pathophysiological mechanism.

Our results are of importance with regard to the need for validation of standardized clinical endpoints for severe disease. Definitions for moderate disease severity have recently attracted more attention. Here we defined moderate disease severity based on the available data in this well-characterized study. In the future, this needs to be further refined for each of the categories to increase the granularity of severity assessment. On the other hand, higher granularity goes with the need for more technical or comprehensive investigations, which might not be available outside of the context of research studies.

## Supporting information

S1 Checklist STROBE checklist. Checklist of items that should be included in reports of cohort studies.
(DOCX)

S1 Table Candidate list of clinical and laboratory variables. The assessment of the percentage non-missing and the percentage abnormal is based on 257 patients with severe leakage or bleeding on the respective first day of severe disease. Linear discriminant analysis (LDA) was performed in 222 patients after excluding individuals with missing values.
(DOCX)

S2 Table Summary of patients with severe bleeding, without evidence of severe organ dysfunction or severe vascular leakage—Or with only moderate vascular leakage. Severe bleeding was classified according to the treating physician and the assessment of the presence of haemodynamic instability. Vascular leakage was assessed daily by clinical assessment (clinical fluid accumulation [FA] like pleural effusion, ascites), and radiologically during the critical period (within 24 hours of defervescence). Radiological evidence was defined as pleural effusion via chest x-ray (CXR) and / or ultrasound (US); or ascites, pericardial effusion.
(DOCX)

S1 Fig Histogram of the discriminant function, with the bars stacked according to the subgroup (bleeding or vascular leakage). The vertical line shows the prediction cut-off. Those meeting the criteria for both subgroups on the same day have been placed in the bleeding subgroup.
(TIF)
