# Supplementary material for: Severe dengue categories as research endpoints—Results from a prospective observational study in hospitalised dengue patients
Source: PLoS Negl Trop Dis. 2020 Mar 4;14(3):e0008076. doi: 10.1371/journal.pntd.0008076 (PMC7055818; doi:10.1371/journal.pntd.0008076)
Supplement: S1 Checklist — Checklist of items that should be included in reports of cohort studies. (DOCX) [file pntd.0008076.s001.docx]

STROBE Statement—Checklist of items that should be included in reports of ***cohort studies***

**S1 STROBE checklist. Checklist for the DENCO study – manuscript on severe dengue categories.**

|  | Item No | Page in manuscript | Recommendation |
| --- | --- | --- | --- |
| **Title and abstract** | 1 | 1-Title /  2-Abstract | 1. Indicate the study’s design with a commonly used term in the title or the abstract   *The prospective nature of the study is indicated in the abstract.* |
|  |  | 2 | 1. Provide in the abstract an informative and balanced summary of what was done and what was found.   *The abstract is a balanced summary of the manuscript.* |
| Introduction | | |  |
| Background/rationale | 2 | 4 | Explain the scientific background and rationale for the investigation being reported.  *The scientific background and the rationale are explained in the introduction / background section.* |
| Objectives | 3 | 4/5 | State specific objectives, including any prespecified hypotheses  *The objectives are stated in the last paragraph of the introduction / background section (lines 105-111).* |
| Methods | | |  |
| Study design | 4 | 5 | Present key elements of study design early in the paper.  *The study design is presented at the beginning of the methods section. We also refer to a previously published article that provides additional information, which is not repeated again in this manuscript.* |
| Setting | 5 | 5 | Describe the setting, locations, and relevant dates, including periods of recruitment, exposure, follow-up, and data collection.  *Methods section, first paragraph, lines 115-124.* |
| Participants | 6 | 5 | 1. Give the eligibility criteria, and the sources and methods of selection of participants. Describe methods of follow-up.   *Additional details can be found in a previous publication of the same data set (cited).* |
|  |  | N/A | (*b*) For matched studies, give matching criteria and number of exposed and unexposed |
| Variables | 7 | 5-6 | Clearly define all outcomes, exposures, predictors, potential confounders, and effect modifiers. Give diagnostic criteria, if applicable  *Methods section, second paragraph of “Study design & patient population”, lines 128-135 & 147-150 (outcomes), methods section, second paragraph of “Statistical Analysis”, lines 150-164 (exposure variables). More details can be found in S1 Table.* |
| Data sources/ measurement | 8* | 6 | For each variable of interest, give sources of data and details of methods of assessment (measurement). Describe comparability of assessment methods if there is more than one group  *Methods section, second paragraph of “Statistical Analysis”, lines 156-164.* |
| Bias | 9 |  | Describe any efforts to address potential sources of bias  *Due to the study design the disease spectrum in the hospitalized patients is biased towards the severe end of the spectrum. However, as we concentrate on this patient subgroup in the manuscript, this does not apply as a specific bias.* |
| Study size | 10 | 5 | Explain how the study size was arrived at  *The study size was originally determined for the revision of the dengue classification. Details can be found in a previous publication (cited).* |
| Quantitative variables | 11 | 6 | Explain how quantitative variables were handled in the analyses. If applicable, describe which groupings were chosen and why  *The methods with regard to the mix of binary and continuous variables are discussed in the Methods section, second paragraph of “Statistical Analysis”, lines 158-164.* |
| Statistical methods | 12 | 6-7 | 1. Describe all statistical methods, including those used to control for confounding   *Methods section, second paragraph of “Statistical Analysis”, lines 153-156 & 164-167.* |
|  |  | 6-7/9 | 1. Describe any methods used to examine subgroups and interactions   *Subgroups: methods section, first paragraph of “Statistical Analysis”, lines 141-143 (stratified by age and continent)*  *Subgroups: methods section, third paragraph of “Statistical Analysis”, lines 168-170 & results section, first paragraph of “Association patterns between clinical and laboratory markers by severity category”, lines 227-229 (linear discriminant analysis comparing severe leakage with bleeding subgroups)* |
|  |  | 6 | 1. Explain how missing data were addressed   *Methods section, second paragraph of “Statistical Analysis”, lines 153-156.* |
|  |  | 5 | 1. If applicable, explain how loss to follow-up was addressed   *Details can be found in a previous publication (cited in Methods section, first paragraph of “Study design & patient population”, line 120).* |
|  |  | 7 | (*e*) Describe any sensitivity analyses  *Methods section, third paragraph of “Statistical Analysis”, lines 170-172: sensitivity analysis was carried out for individuals meeting the criteria for both severe bleeding and severe leakage subgroup.* |
| Results | | |  |
| Participants | 13* | 5 | 1. Report numbers of individuals at each stage of study—eg numbers potentially eligible, examined for eligibility, confirmed eligible, included in the study, completing follow-up, and analysed |
|  |  |  | 1. Give reasons for non-participation at each stage |
|  |  |  | 1. Consider use of a flow diagram   *Methods section, first paragraph of “Study design & patient population”, lines 115-116 & second paragraph of “Study design & patient population”, 125-130, for a-c* |
| Descriptive data | 14* | 7 | 1. Give characteristics of study participants (eg demographic, clinical, social) and information on exposures and potential confounders   *Results section, first & second paragraph of “Epidemiology of severe dengue”, lines 177-188 & Table 3* |
|  |  | Annex | 1. Indicate number of participants with missing data for each variable of interest   *S1 Table* |
|  |  | 5 | 1. Summarise follow-up time (eg, average and total amount)   *The follow-up time was described before. Details can be found in the previous publication (8).* |
| Outcome data | 15* | 7-8 | Report numbers of outcome events or summary measures over time  *Results section, paragraphs of “Severe dengue categories”, lines 190-201 (severe dengue) & paragraphs of “Moderate severity categories”, lines 203-222 (moderate dengue), Table 4* |
| Main results | 16 | 9 | 1. Give unadjusted estimates and, if applicable, confounder-adjusted estimates and their precision (e.g. 95% confidence interval). Make clear which confounders were adjusted for and why they were included   *Results section, first paragraph of “Association patterns between clinical and laboratory markers by severity category”, lines 231-238.* |
|  |  | N/A | 1. Report category boundaries when continuous variables were categorized |
|  |  | N/A | (*c*) If relevant, consider translating estimates of relative risk into absolute risk for a meaningful time period |
| Other analyses | 17 | 9 | Report other analyses done—eg analyses of subgroups and interactions, and sensitivity analyses  *Results section, second paragraph of “Association patterns between clinical and laboratory markers by severity category”, lines 239-244.* |
| Discussion | | |  |
| Key results | 18 | 10-11 | Summarise key results with reference to study objectives  *Discussion section, paragraphs 3-6, lines 268-299.* |
| Limitations | 19 | 12 | Discuss limitations of the study, taking into account sources of potential bias or imprecision. Discuss both direction and magnitude of any potential bias  *Discussion section, last paragraph, lines 322-332.* |
| Interpretation | 20 | 13 | Give a cautious overall interpretation of results considering objectives, limitations, multiplicity of analyses, results from similar studies, and other relevant evidence  *Conclusions, paragraphs 2-3, lines 341-351.* |
| Generalisability | 21 | 13 | Discuss the generalisability (external validity) of the study results  *Conclusions, last paragraph, lines 352-357.* |
| Other information | | |  |
| Funding | 22 | 14 | *Funding section* |

*Give information separately for exposed and unexposed groups.

**Note:** An Explanation and Elaboration article discusses each checklist item and gives methodological background and published examples of transparent reporting. The STROBE checklist is best used in conjunction with this article (freely available on the Web sites of PLoS Medicine at http://www.plosmedicine.org/, Annals of Internal Medicine at http://www.annals.org/, and Epidemiology at http://www.epidem.com/). Information on the STROBE Initiative is available at http://www.strobe-statement.org.
