# Supplementary material for: Severe dengue categories as research endpoints—Results from a prospective observational study in hospitalised dengue patients
Source: PLoS Negl Trop Dis. 2020 Mar 4;14(3):e0008076. doi: 10.1371/journal.pntd.0008076 (PMC7055818; doi:10.1371/journal.pntd.0008076)
Supplement: S1 Table — The assessment of the percentage non-missing and the percentage abnormal is based on 257 patients with severe leakage or bleeding on the respective first day of severe disease. Linear discriminant analysis (LDA) was performed in 222 patients after excluding individuals with missing values. (DOCX) [file pntd.0008076.s002.docx]

**S1 Table. Candidate list of clinical and laboratory variables.**

The assessment of the percentage non-missing and the percentage abnormal is based on 257 patients with severe leakage or bleeding on the respective first day of severe disease. Linear discriminant analysis (LDA) was performed in 222 patients after excluding individuals with missing values.

| **N** | **Name** | **Definition** | **Note** | **Percentage non-missing values** | **Percentage abnormal values** | **Inclusion in analysis (reason)** |
| --- | --- | --- | --- | --- | --- | --- |
| **Clinical variables** | | | | | | |
| 1 | Skin flush | Blanches with pressure |  | 95 | 34 | yes |
| 2 | Rash | Macular non-petechial rash |  | 100 | 33 | yes |
| 3 | Not eating normally | Self-explanatory | Combined into ‘nausea’ | 100 | 73 | yes |
| 4 | Not drinking normally | Self-explanatory |  |  |  |  |
| 5 | Persistent vomiting | Vomiting ≥ 5 times per day |  | 100 | 2.3 | no (< 5% abnormal) |
| 6 | Fainting | Temporary loss of consciousness, with spontaneous recovery |  | 92 | 4.6 | no (< 5% abnormal) |
| 7 | Oedema | Anywhere in the body, this can be peripheral or facial or both |  | 100 | 15 | yes |
| 8 | Clinical ascites | Self-explanatory |  | 100 | 27 | yes |
| 9 | Clinical pleural effusion | Self-explanatory |  | 100 | 37 | yes |
| 10 | Abdominal pain | Abdominal pain severe enough to be the chief complaint of the patient and cause distress | Combined into ‘abdominal pain / tenderness’ | 100 | 72 | yes |
| 11 | Abdominal tenderness | Tenderness elicited on gentle palpation by the doctor. |  |  |  |  |
| 12 | Palpable liver | Self-explanatory |  |  |  | no (because enlarged liver is included) |
| 13 | Enlarged liver | Liver enlarged >= 2cm below coastal margin (by palpation) |  | 100 | 28 | yes |
| 14 | Lethargy | Lack of energy and motivation, expressed in sluggishness or apathy. Also often combined with drowsiness, which is defined with a need to sleep. |  | 100 | 15 | yes |
| 15 | Restlessness | Increased tension and irritability often combined with extreme arousal |  | 100 | 3.9 | no (< 5% abnormal) |
| 16 | Convulsions | Self-explanatory | Combined into ‘neurology abnormal’ | 100 | 2.7 | no (< 5% abnormal) |
| 17 | Meningism | Self-explanatory |  |  |  |  |
| 18 | Coma scale | Glasgow coma scale < 15 / Blantyre coma scale < 5 |  |  |  |  |
| 19 | Bleeding tendency | Any bleeding irrespective of the site including haematuria |  | 100 | 71 | no (skin bleeding only and mucosal bleeding were included) |
| 20 | Minor skin bleeding only | Self-explanatory |  | 100 | 39 | yes |
| 21 | Mucosal bleeding | Bleeding from mucosal membranes |  | 100 | 30 | yes |

| **N** | **Name** | **Definition / Units** | **Percentage non-missing data** | **Percentage abnormal**  ***binary variables*** | **median (interquartile range)**  ***continuous variables*** | **Inclusion in analysis (reason)** |
| --- | --- | --- | --- | --- | --- | --- |
| **Laboratory variables** | | | | | | |
| 1a | Platelet count, continuous | 1000 cells/µl | 94 |  | 39 (22-70) | yes |
| 1b | Platelet count, binary | < 100,000 cells/µl | 94 | 85 |  | yes |
| 2a | White blood cell count, continuous | 1000 cells/µl | 86 |  | 4.1 (2.6-5.7) | no (>10% missing values) |
| 2b | White blood cell count, binary | < 2000 cells/µl | 86 | 9 |  | no (>10% missing values) |
| 3a | Lymphocytes | % of white blood cells | 80 |  | 38 (30-49) | no (>10% missing values) |
| 3b | Lymphocytes, binary | > 50% of white blood cells | 80 | 21 |  | no (>10% missing values) |
| 4a | Haematocrit change, continuous | % change compared to population baseline | 92 |  | 17 (7-28) | yes |
| 4b | Haematocrit change, binary | > 20% change compared to population baseline | 92 | 40 |  | yes |
| 5 | AST | units /l | 65 |  | 127 (78-201) | no (>10% missing values) |
| 6 | ALT | units /l | 65 |  | 66 (36-120) | no (>10% missing values) |
