# Supplementary material for: Severe dengue categories as research endpoints—Results from a prospective observational study in hospitalised dengue patients
Source: PLoS Negl Trop Dis. 2020 Mar 4;14(3):e0008076. doi: 10.1371/journal.pntd.0008076 (PMC7055818; doi:10.1371/journal.pntd.0008076)
Supplement: S2 Table — Severe bleeding was classified according to the treating physician and the assessment of the presence of haemodynamic instability. Vascular leakage was assessed daily by clinical assessment (clinical fluid accumulation [FA] like pleural effusion, ascites), and radiologically during the critical period (within 24 hours of defervescence). Radiological evidence was defined as pleural effusion via chest x-ray (CXR) and / or ultrasound (US); or ascites, pericardial effusion. (DOCX) [file pntd.0008076.s003.docx]

**S2 Table. Summary of patients with severe bleeding, without evidence of severe organ dysfunction or severe vascular leakage - or with only moderate vascular leakage.**

Severe bleeding was classified according to the treating physician and the assessment of the presence of haemodynamic instability.
Vascular leakage was assessed daily by clinical assessment (clinical fluid accumulation [FA] like pleural effusion, ascites), and radiologically during the critical period (within 24 hours of defervescence). Radiological evidence was defined as pleural effusion via chest x-ray (CXR) and / or ultrasound (US); or ascites, pericardial effusion.

| **Country** | **Age (y)** | **Type of bleeding** | **Lowest (or drop) Hemoglobin** | **Lowest PLT count** | **Blood products** | **Vascular leakage component** |
| --- | --- | --- | --- | --- | --- | --- |
| **No vascular leakage** | | | | | | |
| Philippines | 13 | Severe Nose bleed | 12.1 | 6 | Whole blood | No clinical FA, no pleural effusion by CXR |
| Philippines | 14 | Hematuria, GI and nose bleed | 11.7 | 8 | Whole blood | No clinical FA, no pleural effusion by CXR |
| Philippines | 15 | Hematuria, GI bleed | 14 to 10.3 in one day | 15 | Fresh frozen plasma | No clinical FA, no pleural effusion by CXR |
| Venezuela | 28 | Severe skin (venepuncture site), nose bleed, and GI bleeding | 10.6 | 29 | Cryoprecipitate and platelet transfusion | No clinical FA, no pleural effusion by CXR, no pleural effusion or ascites by US. No respiratory distress. |
| **Moderate vascular leakage (clinical or radiological fluid accumulation without respiratory distress)** | | | | | | |
| Philippines | 19 | Hematuria, GI bleed | 15.1 | 7 | Whole blood | Clin pleural effusion; pleural effusion on CXR, but no resp. distress |
| Philippines | 26 | Severe GI bleeding | 14.5 | 22 | cryoprecipitate | Clin. pleural effusion; pleural effusion in CXR, but no resp. distress |
| Vietnam | 3 | Severe skin bleeding | 11.6 | 27 | No blood products | Ascites and clin. pleural effusion, but no resp. distress |
| Venezuela | 22 | Severe skin bleeding (venepuncture site), also GI and oral cavity | 11.5 | 27 | Platelet transfusion | Clin. pleural effusion; pleural effusion, ascites, and gallbladder wall thickening in US, but no resp. distress |
| **Unclassifiable** | | | | | | |
| Philippines | 22 | Hematuria, GI and nose bleed | 7.9 to 3.4 in two days | 30 | Packed red cells, combination of blood products | No clinical FA, no pleural effusion by CXR (but CXR done 2d after critical period) |
| Brazil | 27 | Severe GI bleeding | 9.2 | 34 |  | No clinical FA, CXR or US not done |
| Brazil | 39 | Hematuria, Nose bleed, venepuncture site | 7 | 32 |  | No clinical FA, CXR or US not done |
| Venezuela | 8 months | Severe skin bleeding | 11 | 55 |  | No clinical FA, CXR or US not done |
