# Supplementary figures and images for: Severe dengue categories as research endpoints—Results from a prospective observational study in hospitalised dengue patients
Source: PLoS Negl Trop Dis. 2020 Mar 4;14(3):e0008076. doi: 10.1371/journal.pntd.0008076 (PMC7055818; doi:10.1371/journal.pntd.0008076)

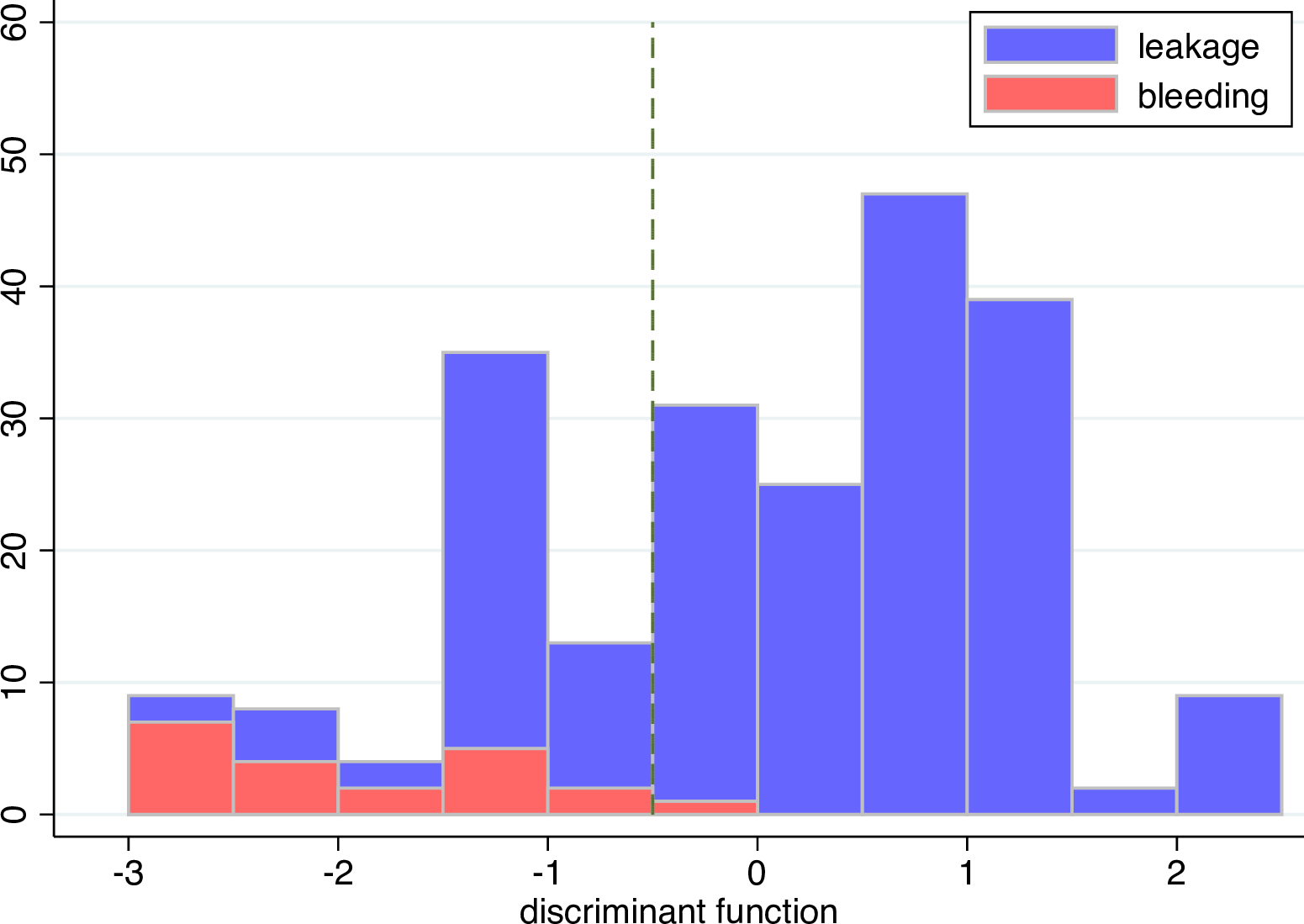

Supplement: S1 Fig — The vertical line shows the prediction cut-off. Those meeting the criteria for both subgroups on the same day have been placed in the bleeding subgroup. (TIF) [file pntd.0008076.s004.tif]
